# Scanner‐agnostic artificial intelligence approach for fast bone scintigraphy

**DOI:** 10.1002/acm2.70709

**Published:** 2026-07-22

**Authors:** Vinicius de Oliveira Menezes, Cleiton Cavalcante Queiroz, Antônio Augusto Silva Oliveira, Aurílio José dos Santos Filho, Allisson Francisco de Morais, Adriano de Oliveira Vigário, Maria Eduarda Flamini, Felipe Alves Mourato, Tsang Ing Ren, Marcos Antônio Dórea Machado

**Affiliations:** ^1^ Department of Diagnostic Imaging, Nuclear Medicine Division Hospital das Clínicas, Federal University of Pernambuco (HC/UFPE–HU Brasil) Recife Pernambuco Brazil; ^2^ Klar Inteligência Artificial Recife Pernambuco Brazil; ^3^ Centro de Informática Universidade Federal de Pernambuco (CIn‐UFPE) Recife Pernambuco Brazil; ^4^ Hospital Universitário Professor Alberto Antunes Universidade Federal de Alagoas (HUPAA–UFAL–HU Brasil) Maceió Alagoas Brazil; ^5^ Núcleo Diagnóstico Maringá Paraná Brazil; ^6^ Hospital Universitário Professor Edgard Santos Universidade Federal da Bahia (HUPES–UFBA–HU Brasil) Salvador Bahia Brazil

**Keywords:** adaptive U‐Net architecture, bone scintigraphy, deep‐learning denoising, dose reduction, image denoising, radiation dose optimization, scanner‐agnostic artificial intelligence

## Abstract

**Purpose:**

Current bone scintigraphy protocols often demand full‐count, 10–15 min scans to preserve image quality, and existing deep‐learning (DL) denoisers typically need to be retrained or retuned for each camera manufacturer. We introduce a scanner‐agnostic adaptive‐diffusion U‐Net designed to reconstruct diagnostic‐grade images from half‐time or half‐dose acquisitions without scanner‐specific retraining.

**Methods:**

A multi‐institutional retrospective set of 3635 studies from four gamma‐camera models was Poisson‐thinned to 10%–70% counts and partitioned for training/validation/testing. The network couples a U‐Net backbone to a trainable isotropic diffusion layer. Quantitative evaluation (SSIM, PSNR, LPIPS) used 182 test scans. Prospective validation in 60 patients, including 20 studies acquired on an unseen Philips BrightView scanner not represented in the training set, was performed, and images were rated by three blinded nuclear medicine physicians on a 5‐point Likert scale. Head‐to‐head comparisons evaluated the diagnostic fidelity of the DL approach.

**Results:**

At 50% counts, DL reconstructions increased SSIM from 0.903 ± 0.045 to 0.963 ± 0.032 and PSNR from 30.50 ± 4.13 dB to 40.85 ± 4.55 dB compared with noisy images, while reducing LPIPS from 0.05 ± 0.01 to 0.03 ± 0.01 (*p* < 0.001). Reader studies showed that double‐speed DL images achieved Likert scores comparable to standard acquisitions (4.4 ± 0.7 vs 4.5 ± 0.5), with no diagnostic discrepancies reported between DL and routine clinical images.

**Conclusion:**

In this multicenter, multi‐vendor study, the adaptive‐diffusion U‐Net effectively supported half‐time, and/or lower‐activity whole‐body bone scintigraphy protocols while preserving diagnostic integrity across scanners and avoiding scanner‐specific retraining, supporting scalable, radiation‐sparing clinical adoption.

## 1 INTRODUCTION

Whole‐body bone scintigraphy is a planar, static nuclear medicine examination that acquires anterior and posterior whole‐body projections, widely recognized as a valuable diagnostic tool due to its high sensitivity, cost‐effectiveness, and ability to provide comprehensive imaging of the entire body in oncologic studies.^1^ In routine adult practice, these exams are most commonly performed with 99mTc‐labeled diphosphonates (e.g. 99mTc‐MDP or equivalent), injected at activities on the order of a few hundred megabecquerels. The procedure involves administering radiopharmaceuticals, resulting in exposure to ionizing radiation; therefore, radiation protection principles must be followed, optimizing radiation doses to ensure diagnostic efficacy and minimize exposure risks. Additionally, scan duration can be extensive, highlighting another opportunity to optimize the throughput and to improve patient comfort. Reducing the radiopharmaceutical dose often increases examination time, highlighting a trade‐off that must be carefully managed.

Bone scintigraphy plays a pivotal role in oncologic imaging, commonly used to detect skeletal metastases in cancers such as prostate, breast, and lung. As with all nuclear medicine procedures, however, it entails exposure to ionizing radiation. International guidelines therefore emphasize adherence to the ALARA (as low as reasonably achievable) principle when determining the radiotracer dose. According to the bone scintigraphy practice guideline of the European Association of Nuclear Medicine (EANM), for adult whole‐body bone scans the recommended reference injected activity is on the order of ∼500 MBq of 99mTc‐labeled diphosphonate, with a typical range of 300–740 MBq.^1^ This administered activity corresponds to an effective dose of roughly 3 to 4 mSv to the patient. The Society of Nuclear Medicine and Molecular Imaging (SNMMI) procedure standards similarly endorse injected activities in the range of about 370–1110 MBq (10–30 mCi) for adults, allowing higher doses for larger patients or specific clinical needs. While these levels are considered safe and diagnostically effective, minimizing the radiopharmaceutical dose remains important—especially for patients who may require multiple follow‐up scans—to reduce cumulative radiation exposure and associated risks over time.^2^ Importantly, any dose reduction must be managed carefully, since lowering the administered activity results in fewer detected photons (counts) and thus noisier images unless compensated by longer acquisition times. This inherent trade‐off between dose and image quality necessitates advanced strategies to optimize bone scan protocols without compromising diagnostic value.

In addition to dose concerns, the practical aspects of scan duration and patient comfort are key factors in whole‐body scintigraphy. Standard protocols for planar bone scans are relatively time‐intensive—for example, using typical table sweep speeds of 10–15 cm/min for delayed whole‐body imaging can translate to 15–30 min or more of acquisition time for a full anterior–posterior study. In practice, many whole‐body bone scintigraphy exams require patients to lie still under the gamma camera for anywhere from about 15 min up to nearly an hour. Such prolonged imaging times can be burdensome. Patients with painful skeletal metastases or other bone pain often find it uncomfortable to remain motionless on the imaging table for extended periods, and elderly or frail patients may struggle to maintain the required position. Even when the procedure itself is painless, having to stay still for long durations can cause significant discomfort and anxiety. Furthermore, lengthy scans limit patient throughput in busy nuclear medicine departments. These clinical and operational constraints are further exacerbated in settings with 99Mo/99mTc supply shortages and limited gamma‐camera capacity, where there is strong pressure to optimize both dose and acquisition time per examination. Reducing scan duration and/or administered activity—while preserving diagnostic integrity—would improve patient experience (by alleviating discomfort and motion‐related issues) and uphold radiation safety principles. This has led to growing interest in innovative techniques, including advanced image reconstruction algorithms and deep‐learning (DL) approaches, to enable faster or lower‐dose bone scintigraphy without loss of diagnostic information.

In recent years, several DL approaches have been proposed to overcome limitations in medical imaging examinations, including image segmentation, denoising, parameter prediction, and optimization of acquisition protocols.^3^, ^4^ Architectures such as U‐Net and adaptive convolutional neural networks demonstrate significant potential in balancing artifact removal and structural detail preservation,^5^, ^6^ enhancing image quality even under reduced‐dose conditions.^7^ Additionally, generative adversarial networks (GANs) and diffusion‐based methods have been applied to nuclear medicine image reconstruction, addressing tasks ranging from noise correction to signal acquisition acceleration.^8^, ^9^ The combined use of these techniques has the potential to maintain diagnostic standards while adhering to radiation protection principles and optimizing imaging protocols.

A major challenge in the clinical implementation of artificial intelligence (AI) models lies in their ability to adapt to variations across scanner manufacturers, acquisition protocols, and imaging settings. Such heterogeneity can significantly limit the generalizability of models trained on data from a single device or patient cohort. To address these challenges, more adaptive DL architectures incorporating diffusion layers have been proposed.^10^, ^11^ For example, U‐Net combined with adaptive diffusion mechanisms has shown strong capability in balancing noise suppression with the preservation of structural details—crucial for maintaining diagnostic accuracy.^12^, ^13^ These strategies potentially enable significant reduction in both image acquisition time and administered radiation dose, without compromising image quality.

Despite these advances, nearly all published DL studies for low‐count bone scintigraphy were tested on a single gamma‐camera^4^, ^5^, ^14^ or systems of the same manufacturer with the same imaging parameters,^6^ which limits real‐world transferability. A recent survey on domain‐generalization in medical imaging identifies this single‐scanner bias as the leading obstacle to clinical translation of AI models.^15^, ^16^ To our knowledge, there is still no validated multicenter, multi‐vendor AI model for planar whole‐body bone scintigraphy that enables at least a 50% reduction in acquisition time and/or administered activity while maintaining diagnostic image quality without scanner‐specific retraining, representing a major unmet clinical need, particularly in scenarios of 99Mo/99mTc shortage and constrained resources.

Importantly, cross‐scanner transfer from a model trained on a single scanner family cannot be assumed. Differences in parameters such as matrix size, pixel size, collimator, detector sensitivity, acquisition speed, and injected activity can alter the image characteristics, leading to domain shift when a model is applied to another vendor or protocol. Thus, a model trained exclusively on specific scanner data might perform well on that scanner family but would not necessarily generalize to other systems without retraining or adaptation.

This paper proposes a DL method to shorten whole‐body bone scintigraphy acquisitions by using adaptive convolutional neural networks. Unlike previous approaches, a diffusion layer is explicitly designed to learn the optimal diffusion coefficients for each clinical scenario and ensure high adaptability. Therefore, we present a scanner‐agnostic architecture with a U‐Net backbone enhanced with adaptive diffusion layers that reconstructs high‐quality images even with lower administered activity and/or reduced scan time, while generalizing robustly across different scanners and settings.

Compared with previous DL approaches for low‐count bone scintigraphy, which have typically relied on single‐manufacturer datasets and scanner‐specific models, this work makes three main contributions. First, we develop a scanner‐agnostic adaptive‐diffusion U‐Net that uses a single set of weights across vendors and protocols, while operating directly at each camera's native matrix size. This design preserves vendor‐specific sampling and resolution characteristics and allows the trainable diffusion layer to adapt its denoising kernels to the noise statistics of each system without any scanner‐specific retraining. Second, we perform a multicenter, multi‐vendor evaluation, including prospective validation on an additional gamma camera that was not seen during training, demonstrating that half‐time acquisitions can preserve diagnostic image quality in routine clinical conditions. Third, we show that the proposed model is computationally lightweight (requiring < 500 MB of GPU memory) and achieves inference times compatible with standard clinical workflows, facilitating deployment on typical hospital hardware and supporting scalable adoption.

## 2 METHODS

### 2.1 Data acquisition

Whole‐body bone scintigraphy images from a total of 3635 retrospective subjects were selected from four gamma cameras across three medical centers (Table 1) for model development. Prospective validation was subsequently performed on three gamma cameras (Philips BrightView, Siemens Symbia, and GE Discovery 630). Collection and use of patient data were approved by the Institutional Review Boards of all participating centers under a multi‐institutional ethics approval protocol. For the retrospective cohort, the requirement for written informed consent was waived because only de‐identified data were used. For the prospective validation cohort, written informed consent was obtained from all participants (or their legal guardians, when applicable) prior to image acquisition and inclusion in the study. Institutional participation consisted of providing data and operational support for clinical performance assessment; no direct contribution was made to the conceptual design or computational implementation of the algorithm. All images and associated metadata were de‐identified in accordance with the HIPAA Privacy Rule and stored on encrypted, access‐controlled servers with full audit logging.

**TABLE 1:** Data distribution by manufacturer and image settings.

Cohort	Manufacturer	Model	Matrix size	Pixel size (row × column, mm)	Subjects (*n*)	Scan speed (cm/min)	Injected activity	Total counts (×10^6^), median (IQR)^a^	Counts/cm^2^ (full image), median (IQR)^b^
Retrospective	GE medical systems	Millennium MG	512 × 128	4.5200 × 4.5200	2465	10	740–925 MBq (20–25 mCi)	2.64 (2.07–3.41)	98.53 (77.40–127.46)
Retrospective	GE medical systems	Discovery 630	1024 × 256	2.4020 × 2.4020	390	12	740–925 MBq (20–25 mCi)	4.19 (3.05–5.23)	138.51 (101.11–173.06)
Retrospective	Siemens NM	Symbia	1024 × 256	2.3976 × 2.3976	710	25	740–1110 MBq (20–30 mCi)	3.71 (3.38–4.67)	123.08 (112.10–155.04)
Retrospective	Siemens NM	e.cam	1024 × 256	2.3976 × 2.3976	70	25	740–1110 MBq (20–30 mCi)	2.44 (1.87–3.62)	80.80 (62.07–120.20)
Prospective clinical evaluation	Philips Medical Systems	BrightView	1024 × 512	2.7950 × 2.7950	20	15	740–925 MBq (20–25 mCi)	2.33 (1.99–2.77)	56.89 (48.58–67.74)
Prospective clinical evaluation	GE Medical Systems	Discovery 630	1024 × 256	2.4020 × 2.4020	20	12	740–925 MBq (20–25 mCi)	4.15 (3.01–5.45)	123.45 (103.36–181.24)
Prospective clinical evaluation	Siemens NM	Symbia	1024 × 256	2.3976 × 2.3976	20	25	740–1110 MBq (20–30 mCi)	3.45 (3.25–4.81)	118.87 (107.51–168.14)

^a^ Total counts (×10^6^) = sum of pixel values in the full‐count reference image; values reported as median and interquartile range (IQR).

^b^ Counts/cm^2^ were calculated from the full image field of view at the original/full‐count level (100%). The last three rows correspond to the prospective validation cohorts.

Within each scanner cohort, examinations followed the corresponding institutional routine acquisition and processing workflow for that system. For whole‐body bone scintigraphy, the institutional protocols specified delayed imaging approximately 3 to 4 h after radiotracer administration. However, radiologist reports and medication‐administration records were not exported with the de‐identified DICOM datasets, and injection‐time fields were not consistently populated in the image headers. Therefore, the injection‐to‐imaging interval could not be retrospectively quantified for most cases. We acknowledge that modest uptake‐time variability may occur in routine practice; however, because imaging was routinely performed within the standard delayed‐imaging window, residual timing variability was expected to have a secondary effect compared with scanner‐ and protocol‐related factors.

Clinical indications for whole‐body bone scintigraphy included staging and restaging of malignant disease (predominantly prostate, breast and lung cancer), evaluation of unexplained bone pain, and assessment of suspected skeletal metastases or multifocal osteoblastic lesions. Only adult patients (≥ 18 years) imaged with standard planar whole‐body protocols were considered. Examinations were excluded if they presented incomplete whole‐body coverage (e.g. missing anterior or posterior projections), severe motion or technical artifacts that rendered the study nondiagnostic, nonstandard acquisition, or reconstruction protocols, or missing DICOM metadata required for harmonized processing (e.g. incomplete pixel data or acquisition parameters). Follow‐up scans performed within the same day and purely postoperative control studies with highly localized fields of view were also excluded to ensure a homogeneous cohort of routine whole‐body bone scintigraphy exams.

### 2.2 Preprocessing

To emulate acquisitions performed at reduced count levels, we generated low‐count versions of each routine whole‐body bone scintigraphy study by applying Poisson‐based subsampling and Monte Carlo count‐reduction simulation^5^ directly to the original DICOM images. For each patient and scanner, the clinically acquired planar whole‐body image was treated as the full‐count reference (100%). Low‐count realizations at 10%, 30%, 50%, and 70% of the original counts were produced by independently sampling each pixel value $N_{full}$, from a binomial distribution $Bin(N_{full},\;p)$, with 𝑝 ∈ {0.10, 0.30, 0.50, 0.70}. For typical count levels, this binomial thinning is mathematically equivalent to drawing from a Poisson distribution with mean $p\;\cdot \;N_{full}$, thus mimicking shorter acquisitions with proportionally fewer detected photons while preserving the underlying system response and spatial resolution. The procedure was implemented in a fully vectorized fashion and applied either to 2D arrays (single planar projections) or to 3D stacks (multi‐frame planar images), depending on the dimension of the pixel data. Apart from the pixel data, the original DICOM headers were kept unchanged, except for the *SeriesDescription* tag, which was updated to label each simulated image according to its relative count level (e.g. “whole‐body 10%,” “whole‐body 30%,” “whole‐body 50%,” “whole‐body 70%”). All subsampled images were stored as new DICOM files in dedicated study folders, preserving the original *SeriesInstanceUID* to allow exact one‐to‐one pairing between full‐count and low‐count images for training and evaluation.

Data were then split into training (90%), validation (5%), and test (5%) sets using a fixed random seed. Intensities were normalized within nonzero masks, inserting $\varepsilon =1\times 10^{-6}$ in empty pixels to avoid numerical instabilities and log artifacts. To address dataset imbalance, such as the predominance of GE Millennium MG images (67.8%), we applied targeted data augmentation (random rotations, flips, and modest intensity scaling) to underrepresented scanner types and employed weighted sampling during training to further reduce sampling bias. While the dataset remained imbalanced in absolute numbers, the combination of these strategies, together with the adaptive diffusion layer's robustness to scanner‐specific variability, effectively mitigated performance degradation across manufacturers and improved overall model generalizability without enforcing strict dataset balancing.

### 2.3 Model building

#### 2.3.1 Network architecture

**FIGURE 1:**
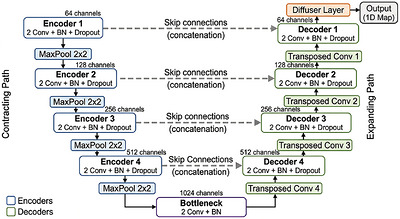
Schematic representation of the proposed U‐Net‐based architecture in a compact up‐down layout. The contracting path performs progressive feature extraction through encoder blocks followed by max‐pooling. The bottleneck aggregates high‐level features, and the expanding path reconstructs the representation through up‐convolution and skip connections. The proposed Diffuser Layer is applied after the final decoder block to refine the representation before generating the output 1D map. Numbers indicate the number of feature channels.

The proposed model employs a 2D U‐Net–like encoder–decoder architecture with skip connections and a custom adaptive diffusion layer (Figure 1).

Each input sample corresponds to a normalized whole‐body planar image, in which anterior and posterior projections are treated independently and combined as a two‐channel 2D array. Images from all scanners were fed to the network at their native resolution (e.g., 512 × 128 and 1024 × 256), without any resizing or cropping, leveraging the fully convolutional nature of the architecture. This design allows the model to operate frame‐wise on variable matrix sizes while preserving the original spatial sampling and scanner‐specific characteristics.

The encoder comprises a sequence of down‐sampling blocks, each containing two 2D convolutions with 3 × 3 kernels, rectified linear unit (ReLU) activations and batch normalization, followed by 2 × 2 max‐pooling. The number of feature channels doubles after each pooling operation, allowing the network to progressively capture larger receptive fields and more abstract representations of the uptake patterns. The decoder mirrors this structure using transposed convolutions for up‐sampling, concatenation with the corresponding encoder feature maps (skip connections), and two additional 3 × 3 convolutions with ReLU and batch normalization per level. A final 1 × 1 convolution maps the decoder output to a single‐channel image representing the predicted full‐count planar scintigraphy.

Several alternative architectures (including standard U‐Net and GAN‐based models) were explored during model development but are not reported in detail here, as a formal baseline comparison was beyond the scope of this work. Training was performed using the Adam optimizer with an initial learning rate of 1 × 10^−^^3^ and a batch size of 1, accommodating variable image dimensions from different scanners. The model was trained for 100 epochs with early stopping triggered after 10 epochs without improvement in the validation loss. The implementation was carried out in Python 3.12.7 using TensorFlow 2.18.0, Keras 3.6.0, pydicom 3.0.1, NumPy 1.26.4, Pandas 2.2.3, scikit‐learn 1.5.2, and an NVIDIA RTX 6000 Ada GPU.

#### 2.3.2 Trainable diffusion layer

On top of the U‐Net decoder, we append a trainable diffusion layer implemented as a depthwise separable 2D convolution with a small kernel K (e.g. 3 × 3). To enforce diffusion‐like behavior, the kernel weights are constrained to be nonnegative and normalized to sum to one. Let x denote the U‐Net output and k(u,v) the normalized kernel coefficients. The final prediction y at pixel (i, j) is given by $y\left(i,\;j\right)=\sum_{\left(u,\;v\right)}k\left(u,\;v\right)\;\cdot \;x\left(i\;-\;u,\;j\;-\;v\right)$, with $\sum_{\left(u,\;v\right)}k\left(u,\;v\right)=1$.

This adaptive diffusion layer performs a local, data‐driven smoothing that selectively suppresses high‐frequency noise while preserving edges and extended skeletal structures. Because the diffusion kernel is learned jointly with the rest of the network, the model can automatically adapt its smoothing behavior to different scanners and acquisition conditions, which is essential for achieving scanner‐agnostic performance.

#### 2.3.3 Loss function

The network is trained end‐to‐end to minimize a composite loss that balances pixel‐wise fidelity, structural similarity and distributional agreement between the predicted image (y) and the full‐count reference image (y_ref). The total loss is defined as:

$$\begin{array}{cc} L & =\alpha \;\cdot \;L_{Huber}\left(y,\;y_{ref}\right)+\beta \;\cdot \;\left(1\;-\;SSIM\left(y,\;y_{ref}\right)\right)\\ & \;+\gamma \;\cdot \;D_{KL}\left(p_{y}\;∥\;p_{y_{ref}}\right), \end{array}$$

where L_Huber is the Huber regression loss computed over all body pixels, SSIM(·, ·) is the structural similarity index measure,^17^ and D_KL(· ∥ ·) denotes the Kullback–Leibler divergence between the normalized intensity histograms *p_y* and *p_y_ref*. The composite loss function weights (Huber loss, α = 0.5; SSIM loss, β = 0.3; KL divergence, γ = 0.2) were determined through iterative hyperparameter tuning informed by both the literature on DL‐based image denoising and internal pilot experiments. Candidate weight combinations were first selected by an experienced medical physicist based on quantitative performance on a validation subset (SSIM, PSNR, and LPIPS). Subsequently, a small set of validation cases was visually reviewed by three nuclear medicine physicians, who qualitatively assessed noise texture, edge preservation, and perceived diagnostic confidence. Among the candidate loss‐weight configurations, the final setting was selected by jointly considering the quantitative image‐quality metrics (SSIM, PSNR, LPIPS) and the qualitative reader impressions, favoring solutions that avoided over‐smoothing, artificial or “hallucinated” uptake patterns, and other visually unfamiliar artifacts while preserving diagnostically relevant uptake patterns comparable to the originals.

### 2.4 Image quality assessment

#### 2.4.1 Model performance

The model's performance was first evaluated on the retrospective test dataset (*n* = 182) using the SSIM,^17^ the Peak Signal‐to‐Noise Ratio (PSNR),^18^, ^19^ and the Learned Perceptual Image Patch Similarity (LPIPS) metric.^20^ This held‐out test set was proportionally derived from the full retrospective cohort and preserved the scanner distribution of the overall dataset, comprising 123 GE Millennium MG, 20 GE Discovery 630, 36 Siemens Symbia, and 3 Siemens e.cam studies. SSIM measures structural similarity based on luminance, contrast, and structure, with values closer to 1 indicating better agreement with the reference. PSNR compares the maximum signal power to reconstruction noise, with higher values indicating better image quality, and LPIPS evaluates perceptual similarity using deep neural networks, with lower scores indicating closer perceptual similarity to the reference. For each subject and projection, these metrics were computed between the full‐count reference image and both the simulated low‐count (Noisy) and DL‐reconstructed images, and summarized per scanner and per count‐reduction level (10%, 30%, 50%, 70%). The same metrics were additionally calculated for the prospective subset (*n* = 60), including Philips BrightView studies that were not present in the training set, to further assess scanner‐agnostic generalizability.

For each metric and simulated count level (10%, 30%, 50%, 70%), paired differences between noisy and DL‐reconstructed images were first assessed for normality using the Shapiro–Wilk test. When the normality assumption was met, paired Student's *t*‐tests were applied; otherwise, Wilcoxon signed‐rank tests were used. Effect sizes were quantified as Cohen's d for paired samples, calculated as the mean difference (DL − Noisy) divided by the standard deviation of the paired differences. Given the strong a priori hypothesis that DL reconstructions would outperform noisy images, *p*‐values are reported descriptively without formal adjustment for multiple comparisons across count levels; values below *p* < 0.01 were interpreted as providing strong evidence of a difference. For ease of interpretation, tables report the absolute value of Cohen's d.

#### 2.4.2 Clinical evaluation

A blinded qualitative assessment was conducted by three experienced nuclear medicine physicians, each with more than 10 years of clinical experience in whole‐body bone scintigraphy, using 60 prospectively acquired whole‐body studies. Each study included up to three image versions: standard institutional acquisition protocol (Original), double scan speed (Noisy), and double scan speed processed with DL. To further validate the model's scanner‐agnostic generalizability, 20 of these studies were acquired on a Philips BrightView system (a manufacturer and camera model not included in the training set). These images were acquired with a matrix size of 1024 × 512, a mean table speed of approximately 15 cm/min, and injected activities in the range 740–925 MBq (20–25 mCi). The clinical spectrum of the 60 prospective validation patients included oncologic staging, restaging, or follow‐up in 38 cases, evaluation of bone pain or suspected inflammatory/degenerative disease in 12 cases and known or suspected metabolic bone disease in 10 cases. Eight examinations were interpreted as normal or near normal, ensuring representation of both pathological and non‐pathological uptake patterns in the prospective cohort.

For the primary qualitative analysis, individual image versions (Original, Noisy, DL) were anonymized and presented in randomized order. Readers were blinded to acquisition conditions, scanner manufacturer, scan speed, and injected activity. Image quality was evaluated using a five‐point Likert scale based on edge definition, presence of artifacts, and regional uniformity (5 = excellent, 4 = good, 3 = fair, 2 = poor, 1 = very poor).^7^ In a secondary paired review, Original and DL images from the same patient were displayed side‐by‐side, still anonymized with respect to acquisition conditions, to assess whether DL processing introduced any visually apparent image abnormality, artificial feature, or any change in perceived diagnostic interpretation.

Ordinal Likert scores were analyzed using the Friedman test for repeated measures across the three conditions (Original, Noisy, DL), followed by post‐hoc pairwise Wilcoxon signed‐rank tests with Bonferroni correction. Inter‐reader agreement for the Likert scores was quantified using Fleiss’ kappa with 95% confidence intervals. A significance level of *p* < 0.01 was adopted for all qualitative comparisons.

## 3 RESULTS

### 3.1 Quantitative image quality

**FIGURE 2:**
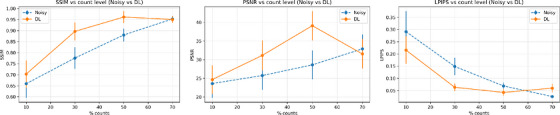
Quantitative image quality as a function of simulated count level in the retrospective test dataset. Mean (± SD) SSIM (left), PSNR (middle) and LPIPS (right) are shown for noisy low‐count images (blue, dashed line) and DL‐reconstructed images (orange, solid line) across all simulated count reductions (10%, 30%, 50% and 70% of the original counts). DL reconstructions consistently increased SSIM and PSNR and reduced LPIPS compared with noisy images at every count level, with the largest gains observed at lower count levels.

The retrospective cohort was randomly partitioned into approximately 90% for training, 5% for validation, and 5% for testing. Comparisons between simulated low‐count (Noisy) and DL‐reconstructed images and their corresponding full‐count clinical references on the held‐out test set (*n* = 182) are summarized in Table 2 and Figure 2. All quantitative values reported in Table 2 refer exclusively to this unseen test set. Across scanners and simulated count‐reduction levels, DL reconstructions showed the clearest quantitative advantage over noisy images at low and intermediate count levels, particularly at 30% and 50%. At 70% counts, where noisy images were already close to the full‐count reference, DL provided limited or no additional quantitative benefit.

**TABLE 2:** Retrospective analysis of image quality metrics across different count levels and reconstruction methods on the held‐out test set (*n* = 182). Values are mean ± SD for structural similarity index (SSIM), PSNR and LPIPS comparing noisy or DL‐reconstructed images with their corresponding full‐count clinical references.

Counts (%)	Condition	SSIM (mean ± SD)	PSNR (dB, mean ± SD)	LPIPS (mean ± SD)
10	Noisy	0.659 ± 0.064	23.61 ± 3.84	0.291 ± 0.085
	DL	0.703 ± 0.062	24.67 ± 3.85	0.216 ± 0.056
30	Noisy	0.776 ± 0.049	25.75 ± 3.84	0.149 ± 0.036
	DL	0.896 ± 0.041	31.14 ± 4.02	0.063 ± 0.015
50	Noisy	0.880 ± 0.029	28.61 ± 3.84	0.069 ± 0.015
	**DL**	**0.961 ± 0.027**	**39.09 ± 3.93**	**0.043 ± 0.014**
70	Noisy	0.953 ± 0.014	32.90 ± 3.84	0.026 ± 0.006
	DL	0.950 ± 0.015	31.54 ± 3.97	0.060 ± 0.017

^a^ Bold characters indicate the best‐performing result among the compared conditions.

At the clinically relevant half‐count scenario (50% counts), DL reconstruction substantially improved image quality compared with noisy images. Mean SSIM increased from 0.880 ± 0.027 for noisy images to 0.961 ± 0.027 for DL reconstructions, PSNR increased from 28.61 ± 3.84 dB to 39.09 ± 3.93 dB, and LPIPS was reduced from 0.069 ± 0.015 to 0.043 ± 0.014 (all *p* < 0.001; Cohen's d in the large range; Table S1). These improvements were observed for both anterior and posterior projections and across all camera models included in the retrospective dataset. Complementary patient‐only analyses excluding background pixels confirmed the same overall trends and are summarized in Tables S2 and S4.

When all simulated count levels were considered (10%–70%), DL outperformed noisy images for SSIM and PSNR at low and intermediate count levels (all *p* < 0.01), with larger gains at the lowest count levels (10%–30%; Table 2, Figure 2). As counts decreased, SSIM and PSNR for noisy images showed a marked degradation, whereas the corresponding DL curves remained closer to the full‐count reference. LPIPS followed the same trend at low and intermediate count levels, with lower perceptual distances for DL reconstructions up to 50% counts (Figure 2). At 70% counts, the limited differences suggest that DL processing had little practical quantitative benefit under near‐standard count conditions, without associated diagnostic discrepancies in the reader study. Taken together, these findings support 50% counts as a practical operating point that offers substantial quantitative gains over noisy images while maintaining a conservative, non‐degrading behavior at near‐standard count levels.

Similar improvements were observed across different camera models and matrix sizes (GE Millennium MG, GE Discovery 630, Siemens Symbia, and Siemens e.cam; 512 × 128 and 1024 × 256; Table S3), supporting the scanner‐agnostic behavior of the proposed model. Although Siemens systems typically operated with shorter acquisition times and lower mean count statistics than GE systems (Table 1), DL reconstructions improved SSIM, PSNR, and LPIPS across manufacturers at low and intermediate count levels. These findings indicate that the network successfully handled variability related to routine acquisition protocols and vendor‐specific imaging characteristics.

**FIGURE 3:**
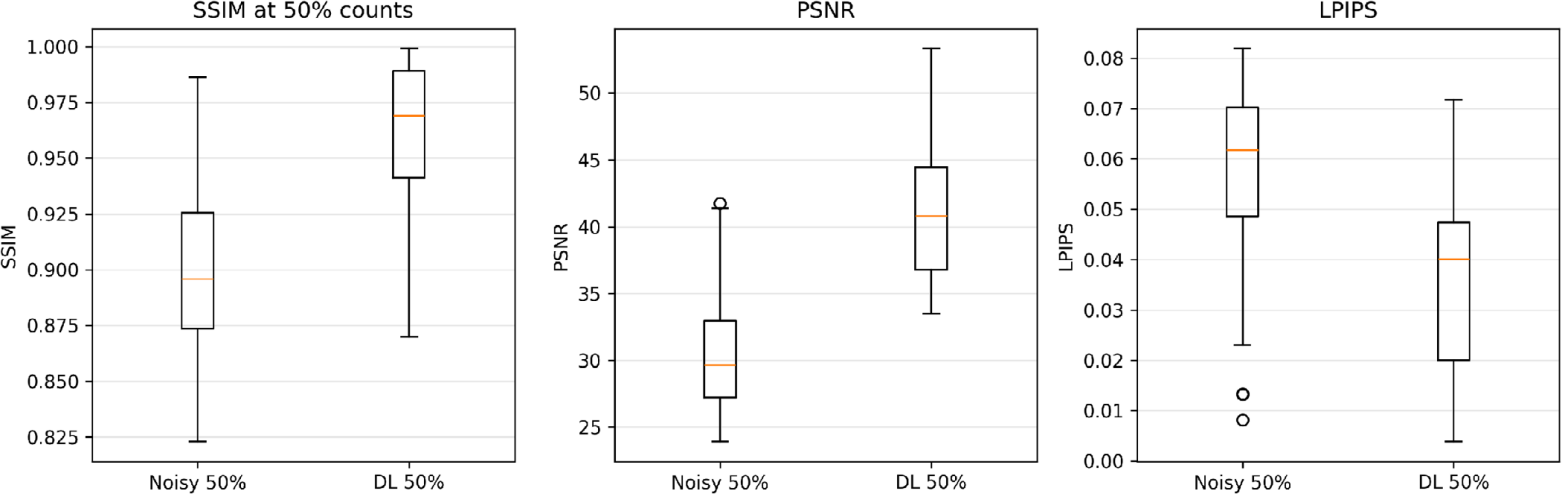
Prospective evaluation of DL reconstructions at half‐count acquisitions. Boxplots of SSIM, PSNR and LPIPS comparing noisy double‐speed acquisitions (Noisy 50%) with DL‐processed double‐speed images (DL 50%) against the corresponding full‐count clinical reference in the prospective test set (*n* = 60). DL reconstructions consistently increased SSIM and PSNR and reduced LPIPS relative to noisy images, confirming the robustness of the model in the prospective setting.

For the prospective dataset (*n* = 60), including 20 studies acquired on a Philips BrightView system not present in the training set, quantitative results again showed that DL‐reconstructed double‐speed acquisitions more closely matched the clinical standard than the corresponding noisy images. This held across all three metrics (SSIM, PSNR, LPIPS), further supporting the scanner‐agnostic generalizability of the proposed approach, as shown in Figure 3, Table 3, and Tables S5 and S6. The corresponding paired statistical comparisons between noisy and DL‐reconstructed images at 50% counts are reported in Table S5, whereas the detailed scanner‐specific prospective metrics are provided in Table S6.

**TABLE 3:** Prospective evaluation of DL reconstruction at 50% counts for different scanner models and matrix sizes. Values are mean ± SD.

Scanner model	Matrix	Condition	SSIM (mean ± SD)	PSNR (dB, mean ± SD)	LPIPS (mean ± SD)
Siemens—Symbia	1024×256	DL	0.955 ± 0.029	39.87 ± 4.30	0.041 ± 0.014
		Noisy	0.887 ± 0.029	30.11 ± 3.91	0.064 ± 0.013
Philips—BrightView	1024×512	DL	0.992 ± 0.006	45.47 ± 3.11	0.014 ± 0.005
		Noisy	0.965 ± 0.010	33.90 ± 3.52	0.028 ± 0.007
GE—Discovery 630	1024×256	DL	0.956 ± 0.028	39.38 ± 3.87	0.042 ± 0.012
		Noisy	0.886 ± 0.025	29.04 ± 3.70	0.064 ± 0.008

A concise overview of the half‐count scenario across both datasets is provided in Table 4. At 50% counts, DL reconstructions consistently improved SSIM, PSNR, and LPIPS relative to noisy images in both the retrospective test set and the prospective cohort, while prospective Likert scores confirmed that double‐speed DL images were qualitatively comparable to routine clinical scans.

**TABLE 4:** Global summary of quantitative and qualitative performance at 50% counts. For both the retrospective test set (*n* = 182) and the prospective cohort (*n* = 60), DL reconstructions at 50% counts substantially improved SSIM, PSNR (dB) and LPIPS relative to noisy images. Prospective Likert scores (1–5) for DL‐reconstructed double‐speed acquisitions were comparable to routine clinical images, with no diagnostic discrepancies reported between DL and standard scans. All paired comparisons between noisy and DL images at 50% counts yielded *p* < 0.001. Detailed per‐vendor and per‐matrix results are provided in Tables S1–S4.

Dataset	Metric	Condition	Mean ± SD	Δ(DL−Noisy)
Retrospective	SSIM	Noisy 50%	0.880 ± 0.027	+0.081
		DL 50%	0.961 ± 0.027	
	PSNR (dB)	Noisy 50%	28.61 ± 3.84	+10.48
		DL 50%	39.09 ± 3.93	
	LPIPS	Noisy 50%	0.069 ± 0.015	−0.026
		DL 50%	0.043 ± 0.014	
Prospective	SSIM	Noisy 50%	0.903 ± 0.045	+0.060
		DL 50%	0.963 ± 0.032	
	PSNR (dB)	Noisy 50%	30.50 ± 4.13	+10.35
		DL 50%	40.85 ± 4.55	
	LPIPS	Noisy 50%	0.050 ± 0.010	−0.020
		DL 50%	0.030 ± 0.010	
Prospective (1–5)	Likert score	Original	4.5 ± 0.5	—
		Noisy 50%	3.4 ± 0.7	1.1
		DL 50%	4.4 ± 0.7	0.1

### 3.2 Qualitative reader study

Table 5 shows the visual image‐quality scores assigned to noisy, DL‐reconstructed, and original images by scanner model and overall. In all clinical scenarios, images acquired at double speed and subsequently processed with DL restored the perceived image quality to a level comparable to that of the current clinical standard. When aggregating all clinical settings, global Likert scores (1–5) were 3.4 ± 0.7 for noisy images, 4.4 ± 0.7 for DL reconstructions, and 4.5 ± 0.5 for original acquisitions. DL images were rated significantly higher than noisy images (p = 1.2 × 10^−^^4^, Cohen's *d* = 1.64) and similarly to original images (DL vs. original: *p* = 0.57, *d* = 0.29). Original images were also rated markedly higher than noisy images (*p* = 5.4 × 10^−^^5^, *d* = 3.16). No diagnostic discrepancies were reported between DL and original images, in agreement with the global summary at 50% counts presented in Table 4.

**TABLE 5:** Mean visual image‐quality scores by scanner for noisy, DL‐reconstructed, and original images. Values are presented as mean ± standard deviation (SD), based on the readers’ qualitative assessment.

Scanner	Noisy, mean (SD)	DL, mean (SD)	Original, mean (SD)
GE Discovery 630	4.0 (0.5)	4.5 (0.6)	4.6 (0.6)
Siemens symbia	3.2 (0.4)	4.5 (0.6)	4.5 (0.5)
Philips brightview	3.3 (0.6)	4.2 (0.6)	4.3 (0.4)
Global	3.5 (0.6)	4.4 (0.6)	4.5 (0.5)

The analysis of image quality issues affecting diagnostic interpretation, namely noise‐related image‐quality degradations such as increased image noise, loss of contrast, and obscuration of subtle focal uptake, revealed that accelerated scans acquired at double speed led to misinterpretation in 6% (4/60) of cases when not processed with DL. In contrast, all images processed with DL yielded diagnostic impressions consistent with those obtained from the standard clinical protocol, with no diagnostic discrepancies reported between DL and original acquisitions.

### 3.3 Representative examples

**FIGURE 4:**
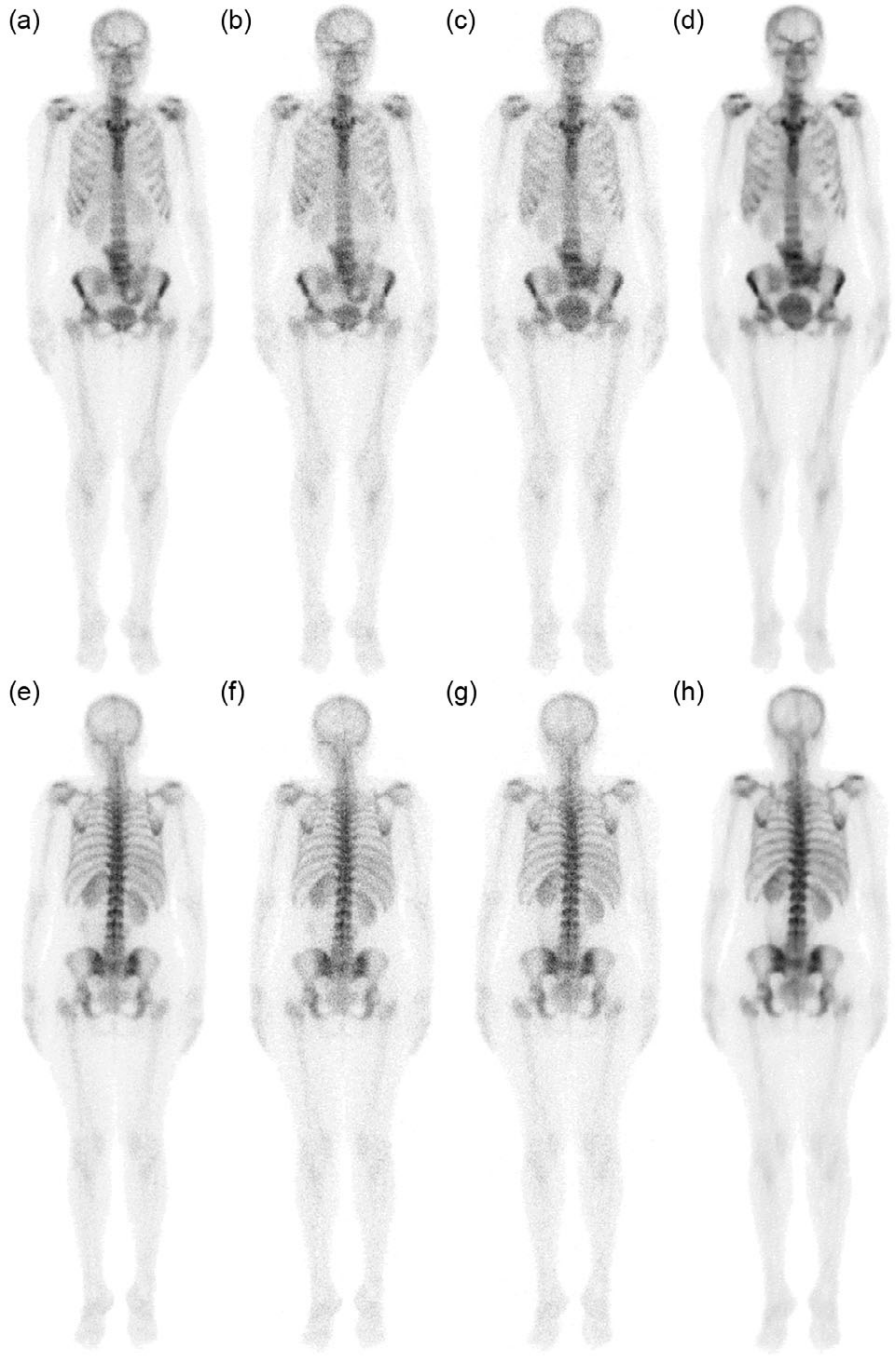
Whole‐body bone scintigraphy scans acquired on a GE Discovery 630 system. Subfigure labels were included directly within the figure to facilitate visual comparison between acquisition and reconstruction conditions. (a, e) Standard institutional acquisition protocol (Original), anterior (a) and posterior (e) views; (b, f) simulated accelerated scan at double speed, anterior (b) and posterior (f) views; (c, g) actual accelerated acquisition at double speed (Noisy), anterior (c) and posterior (g) views; (d, h) accelerated acquisition further processed by deep learning (DL), anterior (d) and posterior (h) views. All panels were displayed using identical grayscale window/level settings to allow fair visual comparison.

**FIGURE 5:**
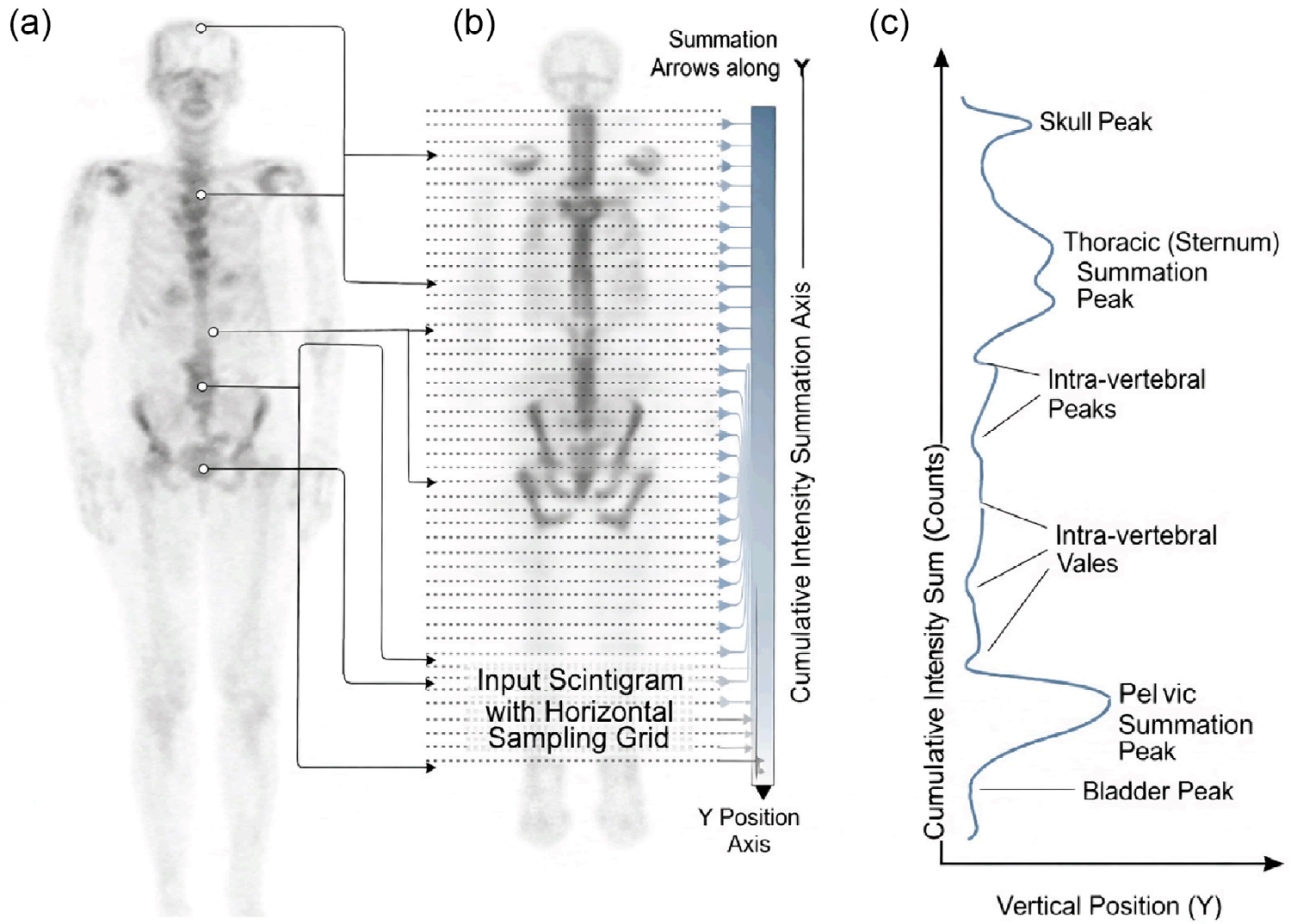
Illustration of the intensity‐sum profile obtained from whole‐body bone scintigraphy. (a) Representative anterior whole‐body scintigraphy image. (b) Schematic representation of the horizontal sampling and summation procedure used to generate the vertical intensity‐sum profile, obtained by summing pixel values along the X‐axis for each Y position. (c) Resulting one‐dimensional cumulative intensity‐sum curve plotted as a function of the vertical position (Y), showing anatomical intensity peaks and valleys, including the skull peak, thoracic/sternal summation peak, intra‐vertebral peaks and valleys, pelvic summation peak, and bladder peak.

**FIGURE 6:**
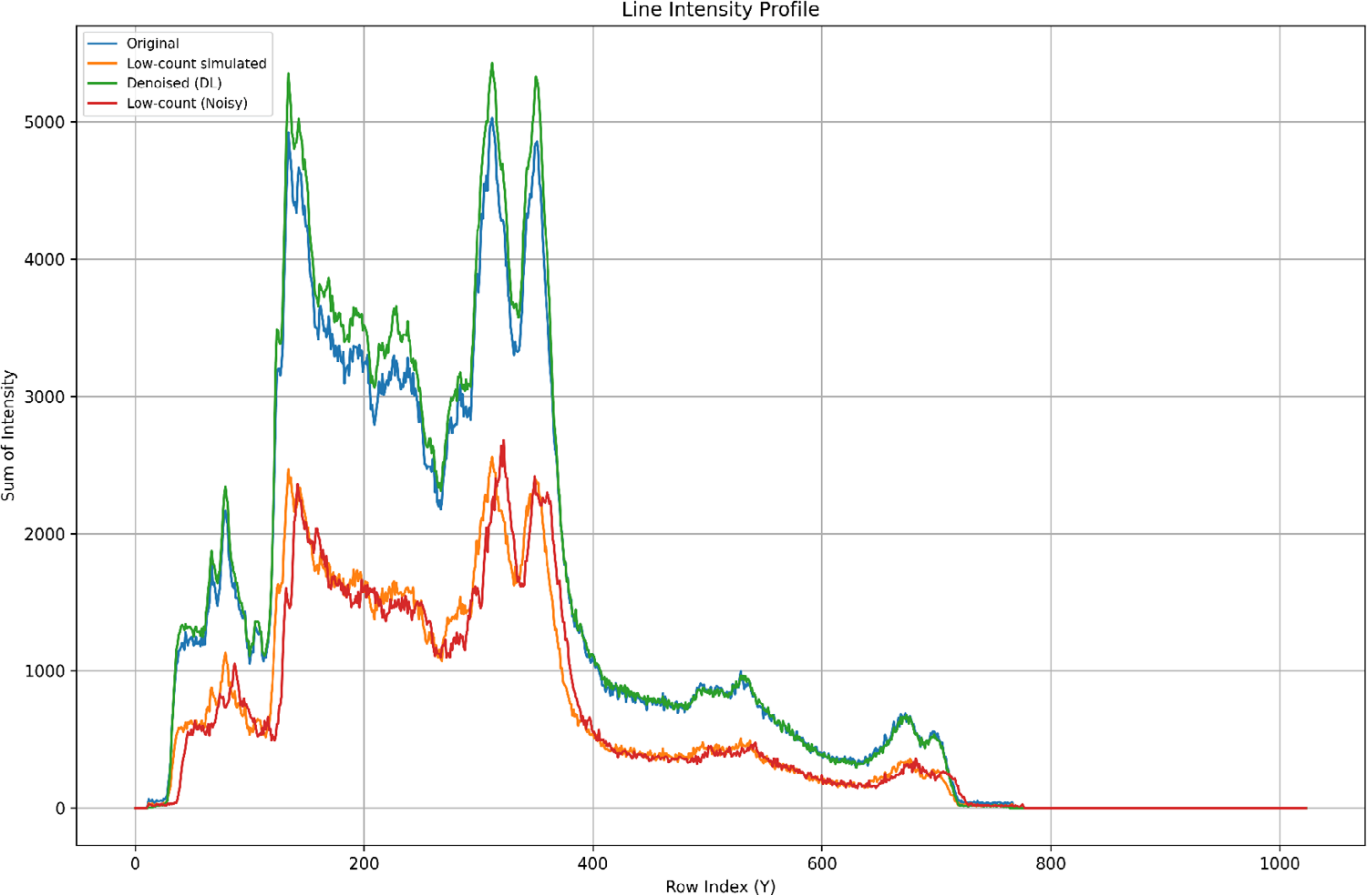
Intensity‐sum profiles for the same whole‐body study shown in Figure 4. Each curve represents the sum of pixel counts along the horizontal axis as a function of the craniocaudal row index (i.e., row‐wise summed intensity). The blue line corresponds to the clinical full‐count reference image (Original), the orange line to the simulated reduced‐count acquisition, the green line to the DL‐reconstructed image, and the red line to the physically acquired noisy low‐count image. The DL profile closely follows the amplitude and shape of the original curve, whereas the noisy profile shows a marked loss of counts and contrast across the field of view. The apparent displacement between the simulated reduced‐count and noisy low‐count curves reflects the fact that these images were obtained at different acquisition times: the simulated low‐count image was generated computationally from the standard acquisition, whereas the noisy image was acquired physically at double table speed. Small differences in patient positioning and/or motion between acquisitions may therefore result in a visible shift in the profile.

Representative examples of the visual improvements achieved with the proposed DL approach are shown in Figures 4, 5, 6. Figure 4 presents whole‐body bone scintigraphy images from a GE Discovery 630 system, comparing the standard clinical acquisition, the simulated reduced‐count image, the actual accelerated noisy acquisition, and the corresponding DL‐reconstructed image, in both anterior and posterior views. Subfigure labels were added directly within the figure to facilitate visual comparison, and all panels were displayed using identical grayscale window/level settings to ensure a fair qualitative assessment. To clarify the derivation of the quantitative profile, Figure 5 schematically illustrates the generation of the intensity‐sum profile by summing pixel values along the X‐axis for each Y position, yielding a one‐dimensional representation of radiotracer distribution along the craniocaudal axis. The corresponding intensity‐sum profiles for the same study shown in Figure 4 are presented in Figure 6, further supporting these qualitative improvements and demonstrating the capability of the DL method to restore signal‐intensity levels comparable to those achieved with the standard clinical protocol. The higher DL peaks observed in some portions of the representative intensity‐sum profile should not be interpreted as systematic count amplification across patients. As previously described, Figure 6 provides a global summary of radiotracer distribution rather than a direct lesion‐level quantification. Therefore, small differences in peak amplitude may arise from changes in image maxima during DL inference, and do not necessarily correspond to increased uptake in a specific anatomical region or to the creation of new focal findings. Importantly, the paired reader assessment did not identify false‐positive diagnostic findings or diagnostic discrepancies between DL‐reconstructed images and standard full‐count acquisitions.

### 3.4 Computational performance

The computational efficiency of the proposed AI model was assessed across simulated count‐reduction levels (10%–70%) using an NVIDIA RTX 3090 (24 GB) and RTX 6000 Ada (48 GB) GPUs, alongside an Intel i9‐14900K CPU. Average inference times were 0.25 s per 1024 × 256 image on the RTX 3090 and 0.17 s on the RTX 6000 Ada, achieving approximately 26× and 40× speed‐ups, respectively, compared to CPU‐only inference (6.27 ± 0.09 s per image). The RTX 3090 required less than 430 MiB VRAM, enabling batch processing (e.g., 10 scans in ∼2.5 s) on mid‐range GPUs with 8–12 GB VRAM. These metrics demonstrate the model's suitability for high‐throughput clinical workflows and compatibility with modest hardware, while remaining operational, though slower, on CPU‐only systems in resource‐limited settings.

## 4 DISCUSSION

Unlike prior DL methods for denoising low‐count bone scintigraphy, which were trained and validated on single‐vendor datasets and often required separate models for each count level, the proposed adaptive‐diffusion U‐Net was trained on a multicenter, multi‐vendor cohort and generalized to an unseen camera model without scanner‐specific retraining. This work presents, to our knowledge, the first multicenter, scanner‐agnostic demonstration that whole‐body bone scintigraphy acquisition time can be effectively halved while preserving diagnostic image quality. By learning a mapping from noisy, double‐speed acquisitions to images that closely resemble the current clinical standard, the model enabled substantial acceleration without compromising diagnostic content. The quantitative improvements in SSIM, PSNR, and LPIPS at the clinically relevant half‐count scenario (50%), combined with the qualitative reader scores and the absence of diagnostic discrepancies between DL and standard images, support the clinical viability of this approach. The complementary quantitative metrics supported the reader‐based findings by capturing structural, intensity, and perceptual similarity between DL reconstructions and full‐count references. As expected, at count levels close to the clinical standard (70%), the gain is minimal or neutral, with no introduction of artifacts. This behavior is consistent with the residual, locally constrained nature of the network: when input image statistics already approximate the reference standard, the model applies only minimal corrections and effectively converges toward an identity mapping. Consequently, high‐count images at 70% are not artificially “hallucinated” or over‐smoothed, but instead exhibit only minor changes relative to the original full‐count reference.

The smoother visual appearance of DL‐reconstructed images should be recognized as a relevant implementation consideration. In the present study, this smoother texture was not associated with diagnostic discrepancies, and DL images achieved Likert scores comparable to standard acquisitions. However, reader acceptance may differ across institutions depending on prior experience and local preferences for image texture. Accordingly, clinical implementation should include reader familiarization with representative DL‐reconstructed cases and local validation before routine adoption.

The heterogeneous nature of the dataset, which included multiple scanners, institutions and acquisition protocols, is both a challenge and a strength of the present study. For example, scans acquired on Siemens systems were generally shorter than those on GE systems, leading to lower average count statistics for Siemens image series, and 67.8% of the training data originated from the GE Millennium MG system. Despite this imbalance, DL reconstructions improved image quality metrics across manufacturers and maintained performance on an unseen Philips BrightView system, suggesting that the adaptive architecture can accommodate routine‐ and manufacturer‐specific variations in practice. By avoiding any resizing and operating directly at the native matrix of each camera, the network preserved vendor‐specific sampling and resolution characteristics while still generalizing across systems.

The present study did not include a dedicated single‐vendor ablation experiment, such as training only on GE Discovery 630 data and testing across other vendors. Therefore, we cannot determine whether a GE‐only model would reproduce the same level of generalizability observed with the multi‐vendor model. However, because scanner‐dependent differences in matrix size, pixel size, detector sensitivity, scan speed, and count density are expected to affect image statistics, the multi‐vendor strategy was intentionally selected to reduce scanner‐specific bias and improve robustness to domain shift.

Total detected counts also varied across camera models (with median summed counts per frame ranging from approximately 2.3 × 10^6^ to 4.2 × 10^6^), reflecting differences in matrix size, detector sensitivity, scan speed and injected activity. Beyond this inter‐scanner variability, the training set deliberately included challenging clinical scenarios such as Paget's disease, diffuse superscan patterns, cases with prominent injection‐site activity and other atypical uptake distributions. These edge cases were further oversampled through targeted data augmentation, so that the network was repeatedly exposed to extreme skeletal uptake and nonphysiological activity patterns. This strategy was intended to reduce overfitting to “typical” presentations and to promote stable, conservative behavior of the model under rare but clinically relevant conditions.

Previous DL studies for low‐count bone scintigraphy have typically relied on single‐manufacturer datasets and, in some cases, required one network per noise level,^4^ limiting generalizability. Related work in other nuclear medicine modalities has explored standard U‐Nets, GANs, SR‐GANs, and alternative convolutional architectures,^21^, ^22^ which can be prone to artifacts, inconsistent texture and overly smooth reconstructions under domain shifts. Although we empirically explored several of these architectures during model development, they frequently exhibited such limitations in our multi‐vendor setting. Rather than exhaustively benchmarking and reporting all these alternatives in the present work, we therefore focused on designing a lightweight adaptive diffusion layer that directly targets these issues while remaining computationally efficient. Motivated by this, we introduced a scanner‐agnostic architecture that pairs a U‐Net backbone with a jointly optimized diffusion layer. Unlike earlier fixed‐kernel CNNs, the proposed layer learns noise‐ and scanner‐specific isotropic kernels on‐the‐fly, combining the flexibility of adaptive kernels^10^ with the efficiency of depthwise separable convolutions already proven in 3D imaging networks.^10^, ^23^ Our results indicate that directly integrating the adaptive diffusion layer endowed the model with strong generalization capacity and robustness across different systems and imaging protocols.

Pure diffusion models have recently been shown to suffer stability issues under worst‐case perturbations,^24^ and critical analyses have questioned their suitability for medical denoising tasks.^25^ The present hybrid CNN + trainable‐diffusion design retains the interpretability of local convolutional filtering while mitigating some of these vulnerabilities, by constraining the diffusion kernel to be normalized, nonnegative, and jointly optimized with the U‐Net backbone. The observed improvements in both quantitative metrics and visual texture, together with the absence of new artifacts in the paired clinical review, are in line with recent reports on PET denoising using related hybrid architectures.^23^

Vendor‐proprietary half‐time or noise‐reduction software may also improve low‐count scintigraphy images and should be considered a relevant alternative. However, such solutions are typically optimized for a specific manufacturer, software version, or reconstruction environment. The main practical distinction of the present approach is its scanner‐agnostic design: a single DL model was trained and validated across multiple manufacturers and settings without scanner‐specific retraining. Therefore, the proposed method is intended as a vendor‐independent framework for heterogeneous scanner installations, including institutions operating equipment from different vendors or older systems without access to proprietary half‐time software.

Recent supply disruptions of molybdenum‐99 (99Mo)—the precursor isotope required to generate technetium‐99 m, the primary radiopharmaceutical used in bone scintigraphy—have created an urgent need for strategies that lower the administered activity without degrading image quality or markedly extending acquisition times.^26^ In this context, scanner‐agnostic AI solutions that support half‐time or half‐dose protocols while preserving diagnostic performance can help safeguard patient access to bone scintigraphy and optimize the use of constrained radiopharmaceutical and scanner resources.

The proposed approach could also be fine‐tuned for other nuclear medicine applications, such as ^1^ ^2^ ^3^I‐MIBG and SPECT imaging, where early studies suggest that DL can enable two‐ to three‐fold faster acquisitions without compromising image quality.^11^ In addition, the lightweight depthwise implementation aligns with emerging Green‐AI guidelines to curb the carbon footprint of medical AI applications,^27^ by enabling deployment on mid‐range GPUs and even CPU‐only systems when necessary. The proposed network is computationally efficient, requiring under 500 MB of GPU memory, and can therefore be deployed on typical clinical GPUs without dedicated high‐end hardware. This combination of scanner‐agnostic behavior, half‐time acquisition capability, prospective multi‐vendor validation, and low computational footprint distinguishes the present work from previous DL denoising approaches and supports its potential for scalable clinical adoption.

## 5 STUDY LIMITATIONS

This study has several limitations. First, the training dataset was imbalanced, with a predominance of GE Millennium MG cases (67.8%) and relatively fewer studies from other systems such as the Siemens e.cam (*n* = 70). Although the adaptive diffusion‐based architecture demonstrated robustness on underrepresented scanners and on the unseen Philips BrightView system, explicit sample balancing or domain‐adaptation strategies were not explored and could further improve performance. In addition, we did not perform a single‐vendor ablation experiment, such as training exclusively on GE Discovery 630 data and testing on other scanners. Such an analysis would be valuable to quantify the incremental benefit of the multi‐vendor training strategy. Second, despite the absence of diagnostic discrepancies between DL and standard images, the qualitative assessment was not fully blinded; paired comparisons may introduce expectation or recall bias, as readers could subconsciously infer differences between images. Future studies employing fully blinded protocols and receiver‐operating‐characteristic (ROC) analyses against an independent reference standard are warranted to more directly quantify diagnostic accuracy. Third, the relatively wide standard deviation in Likert scores (± 0.5–0.7) reflects interobserver variability and the influence of heterogeneous imaging protocols and scanner types on perceived image quality. Fourth, the present model was trained and tested only on standard whole‐body planar bone scintigraphy. Spot views were not included, and therefore generalization to non‐whole‐body scintigraphy applications cannot be assumed without additional dedicated training and validation. Fifth, this study did not include a direct head‐to‐head comparison with vendor‐proprietary half‐time or noise‐reduction implementations. Such comparisons would be clinically valuable to determine whether scanner‐agnostic DL provides equivalent or superior performance relative to manufacturer‐specific solutions under matched acquisition conditions. This was beyond the scope of the present work and should be addressed in future studies. Finally, the limited number of prospective cases per scanner (*n* = 20) increases the impact of individual outlier assessments. Nevertheless, the consistent superiority of DL‐enhanced images over noisy counterparts at low and intermediate count levels, together with the quantitative gains, the complementary use of perceptual (LPIPS) and structural (SSIM/PSNR) metrics, and the low computational footprint, supports the clinical potential of the proposed method. Future studies with explicit domain balancing or few‐shot adaptation may further improve performance on underrepresented vendors.

## 6 CONCLUSION

This study demonstrates that a scanner‐agnostic adaptive U‐Net architecture augmented with a trainable diffusion layer can effectively halve whole‐body bone scintigraphy acquisition time while preserving diagnostic image quality across a heterogeneous, multi‐institutional and multi‐vendor dataset. By avoiding scanner‐specific retraining and maintaining diagnostic agreement between DL‐reconstructed half‐time images and routine clinical protocols, the proposed approach streamlines clinical deployment and supports wider adoption of accelerated or lower‐dose bone scintigraphy, particularly in settings facing 99Mo/99mTc supply constraints and limited scanner capacity.

## AUTHOR CONTRIBUTIONS

Vinicius de Oliveira Menezes contributed to conceptualization, methodology, software, validation, formal analysis, investigation, resources, data curation, writing—original draft, writing—review and editing, visualization, supervision, and project administration. Cleiton Cavalcante Queiroz contributed to methodology, investigation, and resources. Antônio Augusto Silva Oliveira contributed to validation, formal analysis, data curation, and resources. Aurílio José dos Santos Filho contributed to validation, formal analysis, data curation, and resources. Allisson Francisco de Morais contributed to investigation, resources, and supervision. Adriano de Oliveira Vigário contributed to validation, formal analysis, and investigation. Maria Eduarda Flamini contributed to validation, formal analysis, and investigation. Felipe Alves Mourato contributed to validation, formal analysis, and investigation. Tsang Ing Ren contributed to supervision, conceptualization, methodology, software, formal analysis, and investigation. Marcos Antônio Dórea Machado contributed to funding acquisition, project administration, supervision, validation, investigation, writing—original draft, and writing—review and editing. All authors read and approved the final manuscript.

## CONFLICT OF INTEREST STATEMENT

Marcos Antônio Dórea Machado, Cleiton Cavalcante Queiroz, and Vinicius de Oliveira Menezes are researchers affiliated with the Brazilian federal hospital network (HU Brasil) and cofounders of Klar, the company responsible for delivering the AI‐based technology described in this study. HU Brasil contributed to the clinical validation under a formal collaboration agreement that includes provisions for institutional benefit sharing, where applicable. These professional relationships are disclosed in the interest of transparency and do not affect the scientific content or conclusions of the work. All other authors declare no competing interests.

## ETHICAL APPROVAL

The study was approved by the Institutional Review Boards of all participating centers (CAAE: 64455622.0.0000.0049). For the retrospective cohort, the requirement for written informed consent was waived because only de‐identified data were used. For the prospective validation cohort, written informed consent was obtained from all participants or their legal guardians, when applicable. All images and metadata were de‐identified and stored on encrypted, access‐controlled servers with restricted access.

## CONSENT FOR PUBLICATION

All authors agree to publish.

## Supporting information



acm270709‐sup‐0001‐TableS1.docx

acm270709‐sup‐0002‐TableS2.docx

acm270709‐sup‐0003‐TableS3.docx

acm270709‐sup‐0004‐TableS4.docx

acm270709‐sup‐0005‐TableS5.docx

acm270709‐sup‐0006‐TableS6.docx

acm270709‐sup‐0007‐SupMat.docx

## Data Availability

The datasets generated and analyzed during the current study are available from the corresponding author on reasonable request.
