# Supplementary material for: Scanner‐agnostic artificial intelligence approach for fast bone scintigraphy
Source: J Appl Clin Med Phys. 2026 Jul 22;27(8):e70709. doi: 10.1002/acm2.70709 (PMC13389637; doi:10.1002/acm2.70709)
Supplement: Supplementary file 1 — acm270709‐sup‐0001‐TableS1.docx [file ACM2-27-e70709-s005.docx]

**Table S1.** Paired comparisons between noisy and DL-reconstructed images at each simulated count level (10–70%). For each metric, paired differences were tested using either the paired Student’s t-test or the Wilcoxon signed-rank test, according to the Shapiro–Wilk normality test (see Methods). Reported p-values are two-sided.

| Counts (%) | Metric | Noisy (mean) ± (sd) | DL (mean) ± (sd) | ΔDL – Noisy | p-value | Cohen's d | Shapiro–Wilk p-value |
| --- | --- | --- | --- | --- | --- | --- | --- |
| 10 | **SSIM** | 0.659 ± 0.064 | 0.703 ± 0.061 | 0.044 | 2.10e^-60^ | 2.394 | 4.12e^-05^ |
| 30 |  | 0.776 ± 0.048 | 0.896 ± 0.040 | 0.121 | 2.00e^-60^ | 3.644 | 2.94e^-11^ |
| **50** |  | 0.880 ± 0.029 | **0.961** ± **0.027** | 0.081 | 2.00e^-60^ | 3.597 | 1.46e^-11^ |
| 70 |  | 0.953 ± 0.013 | 0.950 ± 0.015 | -0.002 | 4.09e^-13^ | 0.496 | 8.65e^-13^ |
| 10 | **PSNR** | 23.61 ± 3.83 | 24.67 ± 3.84 | 1.07 | 2.00e^-60^ | 10.815 | 2.14e^-11^ |
| 30 |  | 25.75 ± 3.83 | 31.14 ± 4.02 | 5.39 | 2.00e^-60^ | 9.109 | 6.81e^-12^ |
| **50** |  | 28.61 ± 3.83 | **39.09** ± **3.93** | 10.48 | 2.00e^-60^ | 6.373 | 1.93e^-09^ |
| 70 |  | 32.90 ± 3.84 | 31.54 ± 3.96 | -1.36 | 5.60e^-60^ | 1.496 | 1.14e^-09^ |
| 10 | **LPIPS** | 0.291 ± 0.085 | 0.216 ± 0.056 | -0.076 | 2.00e^-60^ | 2.38 | 4.68e^-03^ |
| 30 |  | 0.149 ± 0.036 | 0.063 ± 0.015 | -0.085 | 2.00e^-60^ | 3.31 | 1.50e^-03^ |
| **50** |  | 0.069 ± 0.015 | 0.043 ± 0.014 | -0.026 | 3.94e^-60^ | 2.42 | 1.62e^-09^ |
| 70 |  | 0.026 ± 0.006 | 0.060 ± 0.017 | 0.034 | 2.00e^-60^ | 2.71 | 3.51e^-03^ |
