# Supplementary material for: Scanner‐agnostic artificial intelligence approach for fast bone scintigraphy
Source: J Appl Clin Med Phys. 2026 Jul 22;27(8):e70709. doi: 10.1002/acm2.70709 (PMC13389637; doi:10.1002/acm2.70709)
Supplement: Supplementary file 2 — acm270709‐sup‐0002‐TableS2.docx [file ACM2-27-e70709-s002.docx]

**Table S2.** Paired comparisons between noisy and DL-reconstructed images at each simulated count level (10–70%), using image-quality metrics computed considering only patient pixels (i.e., excluding background pixels outside the patient). For each metric, paired differences were tested using either the paired Student’s t-test or the Wilcoxon signed-rank test, according to the Shapiro–Wilk normality test (see Methods). Reported p-values are two-sided.

| Counts (%) | Metric | Noisy (mean) ± (sd) | DL (mean) ± (sd) | ΔDL – Noisy | p-value | Cohen's d | Shapiro–Wilk p-value |
| --- | --- | --- | --- | --- | --- | --- | --- |
| 10 | **SSIM** | 0.433 ± 0.086 | 0.513 ± 0.096 | 0.080 | 3.20e-223 | 3.734 | 2.55e-26 |
| 30 |  | 0.627 ± 0.069 | 0.844 ± 0.070 | 0.217 | 3.00e-223 | 6.833 | 1.59e-18 |
| **50** |  | 0.804 ± 0.045 | **0.949 ± 0.045** | 0.146 | 3.00e-223 | 6.542 | 1.63e-12 |
| 70 |  | 0.925 ± 0.023 | 0.918 ± 0.023 | -0.007 | 1.06e-147 | -0.846 | 5.04e-20 |
| 10 | **PSNR** | 20.59 ± 3.43 | 21.69 ± 3.44 | 1.110 | 3.02e-223 | 11.348 | 3.15e-40 |
| 30 |  | 22.74 ± 3.44 | 28.29 ± 3.64 | 5.550 | 3.00e-223 | 9.491 | 9.23e-35 |
| **50** |  | 25.62 ± 3.44 | **36.43 ± 3.58** | 10.810 | 3.00e-223 | 6.743 | 1.72e-27 |
| 70 |  | 29.95 ± 3.44 | 28.33 ± 3.53 | -1.620 | 1.75e-222 | -1.662 | 3.14e-20 |
| 10 | **LPIPS** | 0.434 ± 0.089 | 0.317 ± 0.057 | -0.117 | 3.32e-223 | -3.017 | 1.68e-09 |
| 30 |  | 0.218 ± 0.035 | 0.089 ± 0.017 | -0.129 | 3.02e-223 | -4.595 | 1.77e-20 |
| **50** |  | 0.098 ± 0.014 | **0.061 ± 0.017** | -0.037 | 6.57e-221 | -2.585 | 1.83e-15 |
| 70 |  | 0.035 ± 0.006 | 0.087 ± 0.019 | 0.052 | 3.00e-223 | 3.442 | 4.29e-11 |
