# Supplementary material for: Scanner‐agnostic artificial intelligence approach for fast bone scintigraphy
Source: J Appl Clin Med Phys. 2026 Jul 22;27(8):e70709. doi: 10.1002/acm2.70709 (PMC13389637; doi:10.1002/acm2.70709)
Supplement: Supplementary file 3 — acm270709‐sup‐0003‐TableS3.docx [file ACM2-27-e70709-s001.docx]

**Table S3.** Retrospective evaluation of SSIM, PSNR and LPIPS across different scanner models, matrix sizes, count levels and reconstruction methods. Values are mean ± SD.

| **Scanner model** | **Matrix** | **Counts (%)** | **Condition** | **SSIM (mean ± SD)** | **PSNR (dB, mean ± SD)** | **LPIPS (mean ± SD)** |
| --- | --- | --- | --- | --- | --- | --- |
| Siemens - Symbia | 1024×256 | 10 | DL | 0.709 ± 0.061 | 25.61 ± 3.86 | 0.193 ± 0.044 |
|  |  |  | Noisy | 0.680 ± 0.055 | 24.62 ± 3.80 | 0.255 ± 0.060 |
|  |  | 30 | DL | 0.883 ± 0.048 | 31.77 ± 4.15 | 0.063 ± 0.017 |
|  |  |  | Noisy | 0.786 ± 0.046 | 26.74 ± 3.81 | 0.132 ± 0.026 |
|  |  | **50** | **DL** | **0.947 ± 0.032** | **38.87 ± 4.48** | **0.043 ± 0.016** |
|  |  |  | Noisy | 0.882 ± 0.031 | 29.56 ± 3.83 | 0.064 ± 0.012 |
|  |  | 70 | DL | 0.950 ± 0.018 | 32.47 ± 4.01 | 0.057 ± 0.018 |
|  |  |  | Noisy | 0.951 ± 0.016 | 33.78 ± 3.87 | 0.026 ± 0.006 |
| Siemens - e.cam | 1024×256 | 10 | DL | 0.725 ± 0.058 | 25.76 ± 3.91 | 0.198 ± 0.066 |
|  |  |  | Noisy | 0.682 ± 0.058 | 24.69 ± 3.89 | 0.263 ± 0.091 |
|  |  | 30 | DL | 0.906 ± 0.034 | 32.31 ± 4.09 | 0.057 ± 0.019 |
|  |  |  | Noisy | 0.794 ± 0.043 | 26.84 ± 3.89 | 0.138 ± 0.044 |
|  |  | **50** | **DL** | **0.967 ± 0.019** | **40.89 ± 3.93** | **0.037 ± 0.014** |
|  |  |  | Noisy | 0.890 ± 0.026 | 29.71 ± 3.90 | 0.064 ± 0.020 |
|  |  | 70 | DL | 0.956 ± 0.012 | 33.07 ± 3.86 | 0.054 ± 0.018 |
|  |  |  | Noisy | 0.957 ± 0.013 | 34.00 ± 3.92 | 0.024 ± 0.008 |
| GE - MILLENNIUM MG | 512×128 | 10 | DL | 0.666 ± 0.051 | 22.72 ± 3.34 | 0.273 ± 0.028 |
|  |  |  | Noisy | 0.604 ± 0.052 | 21.56 ± 3.34 | 0.382 ± 0.049 |
|  |  | 30 | DL | 0.906 ± 0.025 | 29.39 ± 3.41 | 0.070 ± 0.010 |
|  |  |  | Noisy | 0.740 ± 0.040 | 23.73 ± 3.34 | 0.187 ± 0.016 |
|  |  | **50** | **DL** | **0.977 ± 0.011** | **37.96 ± 3.27** | **0.046 ± 0.012** |
|  |  |  | Noisy | 0.866 ± 0.024 | 26.63 ± 3.34 | 0.082 ± 0.008 |
|  |  | 70 | DL | 0.944 ± 0.010 | 29.03 ± 3.28 | 0.071 ± 0.012 |
|  |  |  | Noisy | 0.951 ± 0.010 | 31.00 ± 3.34 | 0.028 ± 0.003 |
| GE - Discovery 630 | 1024×256 | 10 | DL | 0.723 ± 0.059 | 25.14 ± 3.64 | 0.193 ± 0.046 |
|  |  |  | Noisy | 0.682 ± 0.055 | 24.08 ± 3.61 | 0.255 ± 0.065 |
|  |  | 30 | DL | 0.898 ± 0.043 | 31.70 ± 3.94 | 0.061 ± 0.015 |
|  |  |  | Noisy | 0.792 ± 0.044 | 26.23 ± 3.61 | 0.134 ± 0.028 |
|  |  | **50** | **DL** | **0.959 ± 0.027** | **39.74 ± 3.58** | **0.041 ± 0.014** |
|  |  |  | Noisy | 0.888 ± 0.029 | 29.08 ± 3.62 | 0.064 ± 0.012 |
|  |  | 70 | DL | 0.955 ± 0.015 | 32.37 ± 3.52 | 0.055 ± 0.016 |
|  |  |  | Noisy | 0.955 ± 0.014 | 33.36 ± 3.64 | 0.024 ± 0.005 |
