# Supplementary material for: Scanner‐agnostic artificial intelligence approach for fast bone scintigraphy
Source: J Appl Clin Med Phys. 2026 Jul 22;27(8):e70709. doi: 10.1002/acm2.70709 (PMC13389637; doi:10.1002/acm2.70709)
Supplement: Supplementary file 4 — acm270709‐sup‐0004‐TableS4.docx [file ACM2-27-e70709-s004.docx]

**Table S4.** Retrospective evaluation of SSIM, PSNR and LPIPS across different scanner models, matrix sizes, count levels and reconstruction methods, considering only patient pixels and excluding background pixels outside the body. Values are mean ± SD.

| **Scanner model** | **Matrix** | **Counts (%)** | **Condition** | **SSIM (mean ± SD)** | **PSNR (dB, mean ± SD)** | **LPIPS (mean ± SD)** |
| --- | --- | --- | --- | --- | --- | --- |
| Siemens - Symbia | 1024×256 | 10 | DL | 0.430 ± 0.140 | 22.55 ± 4.01 | 0.272 ± 0.067 |
|  |  |  | Noisy | 0.372 ± 0.124 | 21.56 ± 3.94 | 0.356 ± 0.090 |
|  |  | 30 | DL | 0.771 ± 0.105 | 28.80 ± 4.48 | 0.089 ± 0.026 |
|  |  |  | Noisy | 0.581 ± 0.102 | 23.68 ± 3.95 | 0.186 ± 0.040 |
|  |  | **50** | **DL** | 0.898 ± 0.066 | 35.84 ± 4.62 | 0.062 ± 0.024 |
|  |  |  | Noisy | 0.769 ± 0.068 | 26.50 ± 3.98 | 0.091 ± 0.019 |
|  |  | 70 | DL | 0.901 ± 0.039 | 29.40 ± 4.05 | 0.081 ± 0.026 |
|  |  |  | Noisy | 0.903 ± 0.035 | 30.72 ± 4.03 | 0.037 ± 0.010 |
| Siemens - e.cam | 1024×256 | 10 | DL | 0.506 ± 0.104 | 23.05 ± 4.07 | 0.290 ± 0.066 |
|  |  |  | Noisy | 0.430 ± 0.097 | 21.98 ± 4.04 | 0.383 ± 0.094 |
|  |  | 30 | DL | 0.832 ± 0.065 | 29.61 ± 4.24 | 0.086 ± 0.018 |
|  |  |  | Noisy | 0.629 ± 0.077 | 24.13 ± 4.05 | 0.202 ± 0.040 |
|  |  | **50** | **DL** | 0.940 ± 0.037 | 38.18 ± 4.08 | 0.056 ± 0.016 |
|  |  |  | Noisy | 0.803 ± 0.049 | 27.00 ± 4.06 | 0.095 ± 0.017 |
|  |  | 70 | DL | 0.921 ± 0.023 | 30.36 ± 4.06 | 0.081 ± 0.021 |
|  |  |  | Noisy | 0.922 ± 0.024 | 31.29 ± 4.07 | 0.035 ± 0.007 |
| GE - MILLENNIUM MG | 512×128 | 10 | DL | 0.528 ± 0.060 | 20.93 ± 2.91 | 0.343 ± 0.033 |
|  |  |  | Noisy | 0.440 ± 0.060 | 19.78 ± 2.91 | 0.481 ± 0.051 |
|  |  | 30 | DL | 0.867 ± 0.032 | 27.60 ± 2.97 | 0.090 ± 0.013 |
|  |  |  | Noisy | 0.632 ± 0.047 | 21.95 ± 2.91 | 0.235 ± 0.020 |
|  |  | **50** | **DL** | 0.968 ± 0.015 | 36.12 ± 3.01 | 0.062 ± 0.014 |
|  |  |  | Noisy | 0.810 ± 0.029 | 24.84 ± 2.91 | 0.102 ± 0.009 |
|  |  | 70 | DL | 0.921 ± 0.012 | 27.30 ± 2.87 | 0.091 ± 0.013 |
|  |  |  | Noisy | 0.931 ± 0.013 | 29.22 ± 2.92 | 0.034 ± 0.004 |
| GE - Discovery 630 | 1024×256 | 10 | DL | 0.535 ± 0.089 | 22.74 ± 3.66 | 0.283 ± 0.058 |
|  |  |  | Noisy | 0.469 ± 0.078 | 21.69 ± 3.64 | 0.374 ± 0.085 |
|  |  | 30 | DL | 0.828 ± 0.068 | 29.27 ± 3.93 | 0.091 ± 0.019 |
|  |  |  | Noisy | 0.651 ± 0.065 | 23.83 ± 3.64 | 0.197 ± 0.036 |
|  |  | **50** | **DL** | 0.931 ± 0.043 | 37.27 ± 3.66 | 0.062 ± 0.019 |
|  |  |  | Noisy | 0.812 ± 0.043 | 26.68 ± 3.65 | 0.094 ± 0.016 |
|  |  | 70 | DL | 0.924 ± 0.023 | 29.96 ± 3.63 | 0.083 ± 0.022 |
|  |  |  | Noisy | 0.924 ± 0.022 | 30.96 ± 3.66 | 0.036 ± 0.007 |
