# Supplementary material for: Scanner‐agnostic artificial intelligence approach for fast bone scintigraphy
Source: J Appl Clin Med Phys. 2026 Jul 22;27(8):e70709. doi: 10.1002/acm2.70709 (PMC13389637; doi:10.1002/acm2.70709)
Supplement: Supplementary file 5 — acm270709‐sup‐0005‐TableS5.docx [file ACM2-27-e70709-s007.docx]

**Table S5**. Paired comparisons between noisy and DL-reconstructed images at 50% counts in the prospective dataset. Tests were performed as described in the Methods (paired t-test or Wilcoxon signed-rank test according to normality).

| Metric | Noisy (mean ± SD) | DL (mean ± SD) | Δ (DL − Noisy) | p-value | Cohen’s d | Test used | Shapiro–Wilk p-value |
| --- | --- | --- | --- | --- | --- | --- | --- |
| SSIM | 0.903 ± 0.045 | 0.963 ± 0.032 | 0.060 | <1.8e^-17^ | 2.941 | Wilcoxon signed-rank | 2.65e^-05^ |
| PSNR (dB) | 30.50 ± 4.13 | 40.85 ± 4.55 | 10.352 | <1.8e^-17^ | 5.911 | Wilcoxon signed-rank | 9.08e^-04^ |
| LPIPS | 0.05 ± 0.01 | 0.03 ± 0.01 | -0.021 | <1.8e^-41^ | 2.40 | Paired t-test | 0.232 |
