# Supplementary material for: Scanner‐agnostic artificial intelligence approach for fast bone scintigraphy
Source: J Appl Clin Med Phys. 2026 Jul 22;27(8):e70709. doi: 10.1002/acm2.70709 (PMC13389637; doi:10.1002/acm2.70709)
Supplement: Supplementary file 6 — acm270709‐sup‐0006‐TableS6.docx [file ACM2-27-e70709-s006.docx]

**Table S6**. Prospective evaluation of image quality metrics for deep-learning reconstruction at 50% counts across different scanner models and matrix sizes, compared with the corresponding noisy 50% reconstructions. Values are reported as mean ± standard deviation (SD) for structural similarity index (SSIM), peak signal-to-noise ratio (PSNR) and learned perceptual image patch similarity (LPIPS).

| Scanner model | Matrix | Counts (%) | Condition | SSIM (mean ± SD) | PSNR (dB, mean ± SD) | LPIPS (mean ± SD) |
| --- | --- | --- | --- | --- | --- | --- |
| Siemens - Symbia | 1024×256 | 50 | DL | 0.955 ± 0.029 | 39.9 ± 4.3 | 0.041 ± 0.014 |
| Siemens - Symbia | 1024×256 | 50 | Noisy | 0.887 ± 0.029 | 30.1 ± 3.9 | 0.064 ± 0.013 |
| Philips BrightView | 1024×512 | 50 | DL | 0.992 ± 0.006 | 45.5 ± 3.1 | 0.014 ± 0.005 |
| Philips BrightView | 1024×512 | 50 | Noisy | 0.965 ± 0.010 | 33.9 ± 3.5 | 0.028 ± 0.007 |
| GE Discovery 630 | 1024×256 | 50 | DL | 0.956 ± 0.028 | 39.4 ± 3.9 | 0.042 ± 0.012 |
| GE Discovery 630 | 1024×256 | 50 | Noisy | 0.886 ± 0.025 | 29.0 ± 3.7 | 0.064 ± 0.008 |
