# Supplementary material for: Scanner‐agnostic artificial intelligence approach for fast bone scintigraphy
Source: J Appl Clin Med Phys. 2026 Jul 22;27(8):e70709. doi: 10.1002/acm2.70709 (PMC13389637; doi:10.1002/acm2.70709)
Supplement: Supplementary file 7 — acm270709‐sup‐0007‐SupMat.docx [file ACM2-27-e70709-s003.docx]

Supplementary Tables legend

**Table S1.** Paired comparisons between noisy and DL-reconstructed images at each simulated count level (10–70%). For each metric, paired differences were tested using either the paired Student’s t-test or the Wilcoxon signed-rank test, according to the Shapiro–Wilk normality test (see Methods). Reported p-values are two-sided.

**Table S2.** Paired comparisons between noisy and DL-reconstructed images at each simulated count level (10–70%), using image-quality metrics computed considering only patient pixels (i.e., excluding background pixels outside the patient). For each metric, paired differences were tested using either the paired Student’s t-test or the Wilcoxon signed-rank test, according to the Shapiro–Wilk normality test (see Methods). Reported p-values are two-sided.

**Table S3.** Retrospective evaluation of SSIM, PSNR and LPIPS across different scanner models, matrix sizes, count levels and reconstruction methods. Values are mean ± SD.

**Table S4.** Retrospective evaluation of SSIM, PSNR and LPIPS across different scanner models, matrix sizes, count levels and reconstruction methods, considering only patient pixels and excluding background pixels outside the body. Values are mean ± SD.

**Table S5**. Paired comparisons between noisy and DL-reconstructed images at 50% counts in the prospective dataset. Tests were performed as described in the Methods (paired t-test or Wilcoxon signed-rank test according to normality).

**Table S6**. Prospective evaluation of image quality metrics for deep-learning reconstruction at 50% counts across different scanner models and matrix sizes, compared with the corresponding noisy 50% reconstructions. Values are reported as mean ± standard deviation (SD) for structural similarity index (SSIM), peak signal-to-noise ratio (PSNR) and learned perceptual image patch similarity (LPIPS).
